# Does Printing Orientation Matter in PolyJet 3D Printed Teeth for Endodontics? A Micro-CT Analysis

**DOI:** 10.3390/jfb16120471

**Published:** 2025-12-18

**Authors:** Cláudia Barbosa, Tiago Reis, José B. Reis, Margarida Franco, Catarina Batista, Rui B. Ruben, Benjamín Martín-Biedma, Jose Martín-Cruces

**Affiliations:** 1 Endodontics and Restorative Dentistry Unit, School of Medicine and Dentistry, University of Santiago de Compostela, 15701 Santiago de Compostela, Spain; cbarbosa@ufp.edu.pt (C.B.);; 2 FP-I3ID, FP-BHS, Faculty of Health Sciences, University Fernando Pessoa, 4200-150 Porto, Portugal; 3 RISE-Health, Faculty of Health Sciences, University Fernando Pessoa, Fernando Pessoa Teaching and Culture Foundation, 4200-150 Porto, Portugal; 4 CDRSP, Polytechnic University of Leiria, 2430-028 Marinha Grande, Portugal; margarida.franco@ipleiria.pt (M.F.); catarinadbatista@gmail.com (C.B.); rui.ruben@ipleiria.pt (R.B.R.); 5 Faculty of Engineering, University of Porto, 4200-465 Porto, Portugal; jose.b.reis05@gmail.com; 6 Oral Sciences Research Group, Endodontics and Restorative Dentistry Unit, School of Medicine and Dentistry, University of Santiago de Compostela, Health Research Institute of Santiago de Compostela (IDIS), 15706 Santiago de Compostela, Spain; benjamin.martin@usc.es

**Keywords:** PolyJet, 3D printing, 3D printed teeth, endodontics

## Abstract

This study aimed to identify the optimal printing orientation (X, Y, or Z axis) and positioning of a mandibular molar presenting an isthmus using PolyJet™ technology. The influence of these parameters on dimensional accuracy and on the behavior of 3D-printed teeth (3DPT) during endodontic preparation with ProTaper Gold^®^ system was evaluated. Six groups (XA, XB, YA, YB, ZA, ZB; n = 10) were printed with different axis orientations and distinct isthmus positions relative to the build platform. All samples underwent micro-computed tomography scanning before and after endodontic preparation. Regarding preoperative analyses—canal volume, centroids, and total tooth volume and area—no significant differences were found between groups XA–YA or XB–YB (*p* > 0.05), supporting their comparability. In contrast, groups ZA and ZB differed significantly from all others (*p* < 0.05), failing to meet equivalence required for further comparison, and were therefore excluded. Postoperative evaluation—volume change, centroid displacement, transportation, and unprepared areas—revealed no significant differences between XA–YA and XB–YB. Within the limitations of this study, both printing orientation and position affected the accuracy and repeatability of 3DPT, with positioning exerting the greatest influence, while their behavior towards endodontic preparation remained consistent across orientations.

## 1. Introduction

Three-dimensional (3D) printing is an additive manufacturing technology in which material is deposited sequentially in layers to create a physical object. Since its introduction, multiple 3D printing techniques have been developed, employing a wide range of materials to fabricate 3D models [1]. In recent years, this technology has transformed manufacturing processes, extending beyond industrial applications to areas such as research, development, and engineering prototyping [2]. In the healthcare sector, 3D printing has shown particular promise for medical education and surgical training, as it enables the production of anatomically accurate simulators that provide realistic tactile feedback and enhance procedural skills under near-clinical conditions [3]. The main advantage of 3D printing over conventional manufacturing methods lies in its capacity to produce virtually any geometry, allowing for highly complex and unconstrained designs [4]. Furthermore, it enables the integration and scaling of intricate internal architectures within a single print; however, the fabrication of structures with complex internal geometries still presents significant technical challenges [2,4].

In the PolyJet™ printing process (Stratasys Ltd., Eden Prairie, MN, USA), multiple nozzles move along the XY plane, depositing a liquid, photosensitive resin onto the build platform. Each deposited layer is immediately cured by ultraviolet light. Once a layer is completed, the build platform lowers by one layer thickness along the Z axis, and the process is repeated until the object is fully printed [5,6]. PolyJet™ technology offers high printing resolution, achieving an in-plane XY resolution to 42.3 µm and a layer thickness (Z axis) as low as 16 µm in “High Quality” mode [4,7,8]. When overhangs or hollow structures are present, a removable support material is simultaneously deposited to stabilize the geometry during printing [5,6]. These characteristics make PolyJet™ particularly suitable for medical devices and surgical models prototyping [8]. Despite its precision, this layer-by-layer deposition method may introduce build defects and inconsistencies due to the spreading and bonding behavior of the resin droplets, known as the staircase effect [9,10]. This effect significantly influences surface finish, which is highly dependent on the printed object orientation and other process parameters [10]. The dimensional accuracy and surface quality of PolyJet™-printed objects are influenced by several factors, including raw material properties, printing parameters (e.g., layer thickness, orientation, and curing conditions), as well as post-curing and post-processing steps. Layer thickness, which can be adjusted by the operator, inversely correlates with production time—thinner layers yield higher resolution but require longer printing time. Optimal virtual positioning of the printed object on the build platform is essential to maximize printing efficiency and ensure adequate support material placement without compromising critical surfaces and hollows. Appropriate orientation selection enhances dimensional accuracy, reduces the need for support material, and minimizes manufacturing time and cost [7,11,12]. As with other additive manufacturing methods, objects produced via PolyJet™ exhibit diverse dimensional characteristics and anisotropic mechanical depending on printing orientation. Consequently, the orientation of the printed component relative to the motion of the printhead and build platform can affect the quality and accuracy of the final product [6,12]. Kechagias et al. (2014) demonstrated that dimensional accuracy of external features is primarily influenced by printhead movement and layer thickness, whereas internal diametric dimensions are similarly affected by layer thickness and scaling factors [13].

Extracted human natural teeth (NT) remain the gold standard for ex vivo endodontic research; however, they present substantial limitations for establishing experimentally standardized groups. The inherent anatomical heterogeneity of the root canal system, combined with interindividual factors such as donor age, which influence dentin morphology and mechanical properties, compromises group comparability [14]. When these baseline parameters differ across groups, any detected differences may reflect anatomical variability rather than the experimental intervention itself. This challenge is exemplified by De-Deus et al. (2020), who demonstrated the difficulty of achieving morphologically equivalent groups: from an initial pool of 1708 mandibular incisors, 251 teeth had to be micro-computed tomography (micro-CT) scanned to obtain only two anatomically pair-matched groups of ten specimens, underscoring the difficulty, time, and cost associated with achieving baseline anatomical equivalence that is an essential prerequisite for valid comparative endodontic investigations [15]. 3D printed teeth (3DPT), generated from micro-CT scans of NT, have emerged as a viable alternative for both research and educational purposes. These printed replicas enable the creation of standardized, reproducible samples that facilitate balanced experimental groups [5,16,17]. Despite these advantages, concerns persist regarding the discrepancies between resin-based printed materials and natural dentin, particularly in terms of radiopacity and hardness [18]. Moreover, standardized protocols for the use of 3DPT in endodontic studies have yet to be fully established [19]. Previous studies have evaluated the influence of printing parameters—such as orientation, layer thickness, and material composition—on the accuracy and predictability of printed objects using PolyJet™ and other additive manufacturing technologies. Also, the mechanical behavior of photopolymer components depends strongly on their UV exposure, it is reasonable to expect that build orientation of printed objects could influence the resulting mechanical properties [7,13]. Nevertheless, these investigations often lack the resolution or specific focus required to address the particular demands of 3DPT for endodontic applications. Reis et al. (2024) demonstrated that 3DPT produced using PolyJet™ technology can accurately replicate the anatomy of NT. However, their work was limited to a multi-rooted tooth with three canals and relatively simple morphology, without anatomical variations. The authors also raised the question of whether the orientation of the tooth on the build platform—along the X, Y, or Z axis—could influence 3DPT dimensional accuracy, suggesting that future studies should address this aspect [5].

The first aim of this study was to investigate whether printing orientation (X, Y, or Z axis) and tooth position on the build platform introduce internal anatomical variation in 3DPT produced with PolyJet™ technology. The objective was to determine how these parameters affect external and internal morphological accuracy and repeatability, with the ultimate goal of establishing reliable printing protocols for producing 3DPT suitable for endodontic research. The corresponding null hypothesis stated that no significant differences would be observed among the different axis orientations and positions. The second aim was to evaluate, using Micro-CT, the behaviour of 3DPT printed under different orientation during endodontic instrumentation with the ProTaper Gold^®^ system (Dentsply-Sirona, Fair Lawn, NJ, USA) (PTG). The null hypothesis for this analysis was that printing orientation would not affect the performance or behaviour of 3DPT during root canal preparation.

## 2. Materials and Methods

The study protocol received approval from the Ethics Committee of Fernando Pessoa University (reference FCS/PI 636/24). A priori sample size estimation was conducted using G*Power software version 3.1.9.7 for Windows (Heinrich Heine Universität Düsseldorf, Düsseldorf, Germany), based on data from a previous study assessing shaping ability [20]. The calculation was performed within the t test family, assuming an effect size of 1.79, a significance level (α) of 0.05, and a statistical power (1−β) of 0.95, which indicated a minimum requirement of 16 samples (8 per group) to detect significant intergroup differences. To account for potential specimen loss during the experimental procedures, 10 samples were allocated to each group.

### 2.1. Natural Specimen Selection

An initial pool of 30 extracted human maxillary and mandibular permanent molars, obtained for reasons unrelated to this investigation, was used. Following extraction, the teeth were collected and stored in distilled water until further processing. Standardized radiographs were obtained in mesiodistal and buccolingual directions to verify eligibility. The inclusion criteria comprised teeth with fully developed apices, the absence of root fractures, external or internal resorption, carious involvement in the region of interest, and any previous endodontic treatment. From the initial pool, four teeth met these criteria and were selected, after which endodontic access cavities were prepared. Canal patency was confirmed by introducing a size #10 K-file (Dentsply Sirona, Charlotte, NC, USA) until the file tip was just visible at the apical foramen. The specimens were subsequently scanned using a micro-CT system (Skyscan 1174; Bruker, Kontich, Belgium) at 50 kV and 800 mA, with a 0.25 mm aluminium filter, rotational steps of 0.9° for a total rotation of 180°, a voxel size of 19.60 μm, and an exposure time of 12,000 ms. Image reconstruction was performed using NRecon software (version 1.7.46; Bruker, Kontich, Belgium) with ring artifact correction (value 3), smoothing (value 3), and beam hardening correction (40%). The reconstructed datasets were processed with CTAn software (version 1.20.3.0; Bruker, Kontich, Belgium) to generate stereolithography (STL) files of both the external tooth structure and internal root canal anatomy. The STL models were imported into MeshLab software (version 2021.10; Visual Computing Lab, ISTI-CNR, Pisa, Italy) for qualitative analysis of root canal morphology and selection of the tooth to be replicated. A mandibular first molar presenting two separate roots, one distal canal, and two mesial canals connected by an isthmus was chosen.

**Figure 1:**
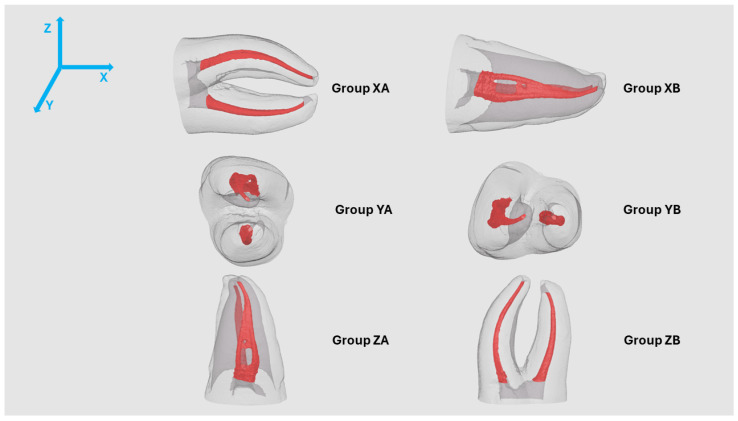
Orientation of the Natural Tooth on the build platform (frontal view) with long-axis parallel to the X, Y and Z Axis and both possible positions of the isthmus.

The selected STL model was fabricated using PolyJet™ technology on a Stratasys Objet30 Prime™ printer (Stratasys Ltd., Eden Prairie, MN, USA) in high-quality mode with a 16 μm layer thickness. The printing materials consisted of RGD525™ high-temperature resin and SUP706B™ support material (Stratasys Ltd.). The tooth was oriented on the build platform with roots long axis aligned parallel to the X, Y, and Z axis, with both possible positions of the isthmus tested, parallel to the build platform or perpendicular to it (Figure 1). Post-printing and before initial micro-CT scan, the support material was removed following the protocol described by Reis et al. [5], obtaining a total of 60 3DPT.

These 60 3DPT were subsequently scanned using the same micro-CT system (Skyscan 1174; Bruker) under the following parameters: 50 kV, 800 mA, rotational steps of 0.9° for a 180° total rotation, voxel size of 19.60 μm, and an exposure time of 5000 ms. Image reconstruction was again performed using NRecon software (version 1.7.46; Bruker), applying ring artifact correction (value 5), smoothing (value 3), and beam hardening correction (50%).

### 2.2. Root Canal Preparation

All procedures were performed by a single operator with 24 years of clinical experience in endodontics and prior clinical expertise with the PTG system. The working length was established by subtracting 1 mm from the measurement obtained when a #10 K-file (Dentsply Sirona) was inserted until its tip became visible at the apical foramen. The 3DPT were stabilized using the ProTrain^®^ system (Simit Dental Srl, Mantua, Italy). Instrumentation was carried out with an X-Smart^®^ Plus electric motor (Dentsply Sirona) set to continuous clockwise rotation, following the manufacturer’s instructions.

### 2.3. Preparation of 3DPT with PTG

A glide path was established using the ProGlider^®^ instrument (Dentsply Sirona), until the working length was reached. Root canal preparation was performed with the PTG system (Dentsply Sirona) in the sequence SX, S1, S2, F1, and F2. Apical patency was verified after the use of each instrument with a size #10 K-file. Each set of instruments was used for the preparation of a single tooth and subsequently discarded. Irrigation was performed between each instrument change using 5 mL of distilled water. Irrigant was delivered with an Irriflex^®^ needle (Produits Dentaires SA, Vevey, Switzerland) and activated using the EndoActivator^®^ (Dentsply Sirona) with a small tip at high frequency for 30 s. Following instrumentation, each canal received two additional irrigation cycles of 5 mL, activated with the EndoActivator^®^, yielding a total irrigation volume of 40 mL per canal. Final drying was achieved with sterile paper points (Dentsply Sirona). After preparation, 3DPT were rescanned using micro-CT under the same scanning and reconstruction parameters as initially established.

### 2.4. Micro-CT Evaluation

Micro-CT analysis was performed by a single examiner, blinded to group allocation. Pre- and post-instrumentation images were superimposed using the three-dimensional registration tool in DataViewer software (version 1.5.6.2; Bruker micro-CT). Quantitative analysis was subsequently conducted with CTAn software (version 1.20.3.0; Bruker micro-CT). The region of interest was defined from the furcation level to the root apex, following the methodology described in a previous study [5].

The changes in centroid position were determined by subtracting pre-instrumentation from post-instrumentation values. The canal transportation was calculated as the vectorial displacement of the centroid coordinates in three dimensions, using the following formula, where “post” represents post-instrumentation and “pre” pre-instrumentation values:

$$\sqrt{{\left(X\mathrm{post}-X\mathrm{pre}\right)}^{2}+{\left(Y\mathrm{post}-Y\mathrm{pre}\right)}^{2}+{\left(Z\mathrm{post}-Z\mathrm{pre}\right)}^{2}}$$

The percentage of unprepared canal surface was calculated as the proportion of unchanged (static) voxels relative to the total number of surface voxels, values were obtained by subtracting the scores of the prepared canals from those of the unprepared ones, and the resulting differences were then converted to percentages. Total tooth volume and total tooth area were calculated after digitally fulfilling the canals using CTAn software (version 1.20.3.0; Bruker micro-CT).

### 2.5. Statistical Analysis

All statistical analyses were conducted using SPSS software, version 30.0 (IBM Corp., Armonk, NY, USA). The normality of quantitative data distributions was assessed using the Shapiro–Wilk test. As the assumption of normality was met, intergroup comparisons were performed using one-way analysis of variance (ANOVA). When overall differences reached statistical significance, pairwise contrasts were examined using Tukey’s post hoc procedure. Comparisons between two unrelated groups were assessed with an independent-samples t test. Data are reported as mean and standard deviation (SD), together with the corresponding median and range values. Statistical significance was established at a *p*-value < 0.05. Boxplots were generated to visually represent data distribution and variability among the 3DPT groups and relative to the NT. For each printed group, absolute and relative mean biases were calculated against the NT by subtracting NT values from the corresponding 3DPT measurements. Agreement between NT and each group was further assessed using Bland–Altman analysis, from which the mean bias and 95% limits of agreement (LoA) (LoA = mean bias ± 1.96 × SD) were computed for all variables. The coefficient of variation (CV) was calculated to assess the relative variability, using the ratio of the SD to the mean (CV = SD/mean × 100).

## 3. Results

**Figure 2:**
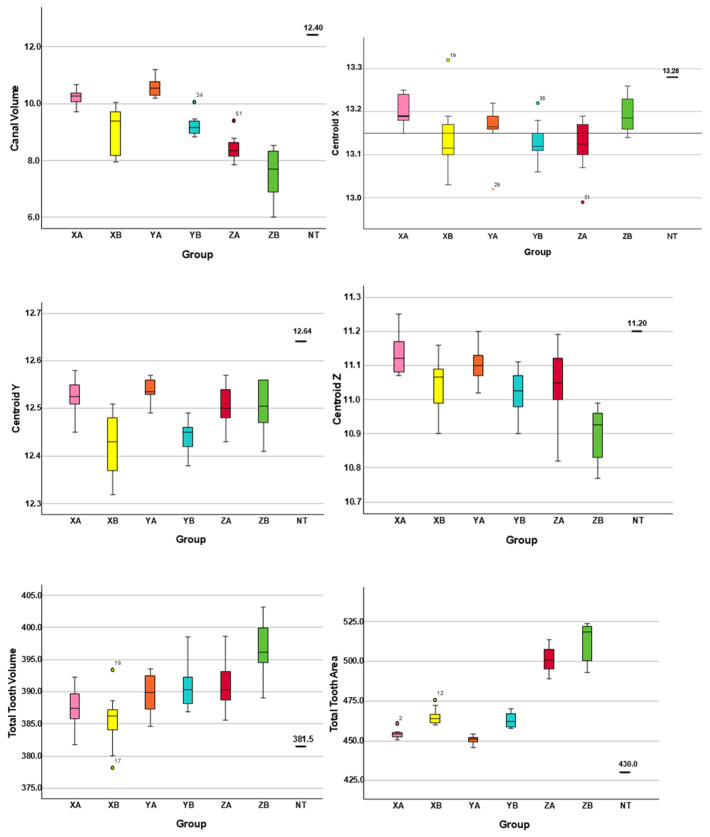
Data distribution and variability among the 3D-printed teeth groups (XA, XB, YA, YB, ZA and ZB) and relative to the natural tooth (NT).

**Figure 3:**
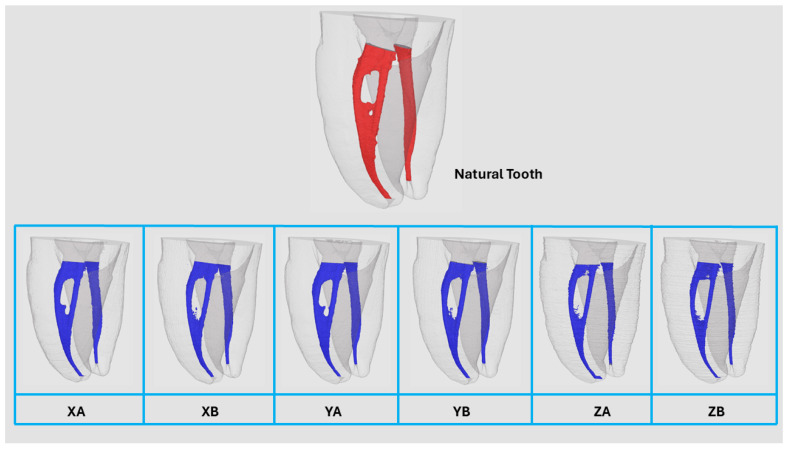
Representative 3D reconstruction of micro-computed tomography scans of samples from each 3D-printed teeth group.

The measurements of canal volume, centroids (X, Y, and Z) before preparation, total tooth volume, and total tooth surface area for the NT and 3DPT groups are presented in Table 1. Statistically significant differences were observed between the NT and 3DPT for all analyzed variables (*p* < 0.05). Data distribution and variability among groups are visually illustrated using boxplots (Figure 2). Figure 3 shows representative 3D reconstruction of micro-CT scans of samples from each 3DPT group.

Table 2 shows the absolute and percentage biases and their 95% LoA for all groups. Overall, the printed models exhibited negative biases for canal volume and centroid coordinates and positive biases for total tooth volume and area. The 95% LoA widened progressively in the Z-axis orientations and were consistently broader in all B positions than in the corresponding A positions.

**Figure 4:**
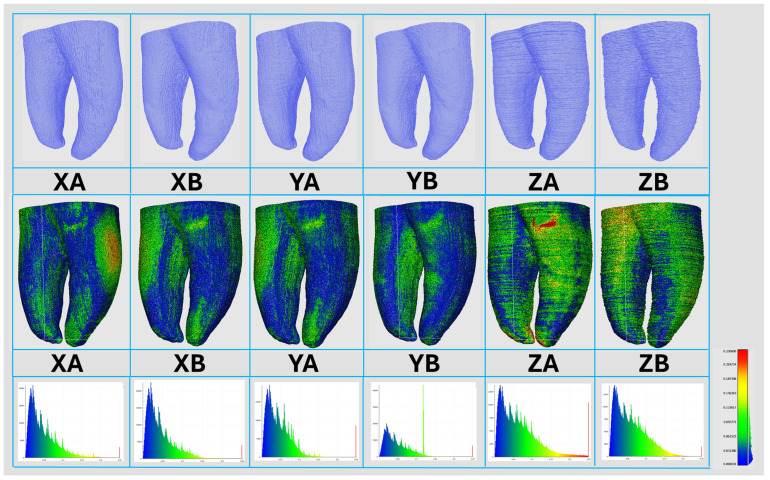
Representative micro-computed tomography reconstructions showing, for each printing group, the surface staircase effect (**top panel**), the corresponding 3D deviation map (**middle panel**), and the histogram of deviation values (**bottom panel**).

Figure 4 illustrates the staircase effect observed on the surface of the 3DPT, the corresponding 3D deviation map and the histogram of deviation values (bottom panel across the different groups).

The CV was calculated to assess the relative variability of total tooth volume and surface area measurements, as shown in Table 3. Groups XA and YA exhibited the lowest variability, with CV values below 1 for both parameters, indicating greater printing consistency.

Intergroup comparison required the absence of statistically significant differences in the four baseline variables—canal volume and centroid coordinates (X, Y, Z). This criterion ensured that the groups started from equivalent anatomical conditions, allowing all observed changes in canal volume, transportation, and untouched areas to be interpreted as resulting exclusively from endodontic preparation. This condition was fulfilled only between groups XA and YA, and between groups XB and YB. In contrast, groups ZA and ZB showed significant differences relative to all other groups and between each other; therefore, they were excluded from further analyses.

**Figure 5:**
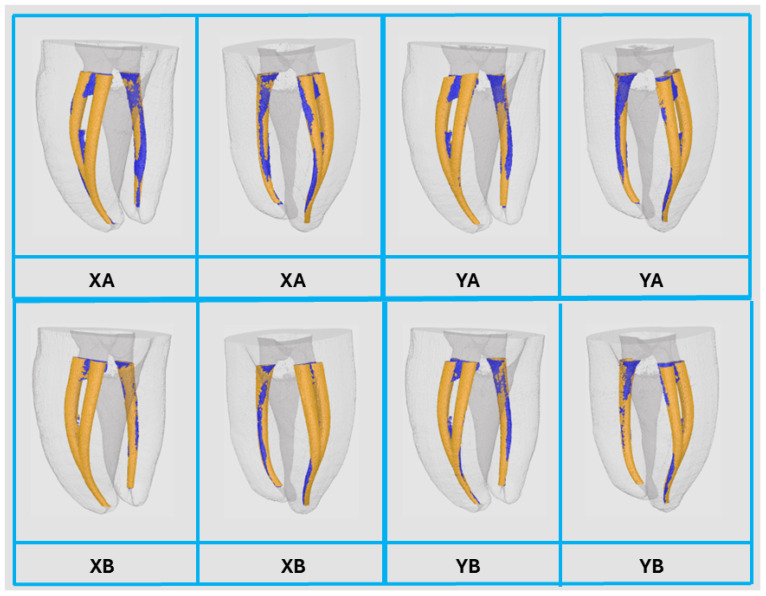
Representative 3D reconstruction of micro-CT scans before (blue) and after (orange) preparation of groups XA versus YA, and XB versus YB, from different views.

A comparative analysis between 3DPT group XA versus YA and group XB versus YB before and after canal preparation with the PTG system is summarized in Table 4. No statistically significant differences (*p* > 0.05) were detected between the corresponding groups for any variable after preparation. The 3DPT exhibited consistent behavior across comparison groups following preparation, with no statistically significant differences (*p* > 0.05) in canal volume, centroid displacement (X, Y, Z), percentage of volume increase, canal transportation, or the percentage of unprepared canal areas (Figure 5).

## 4. Discussion

The first aim of this study evaluated whether the orientation in which a tooth is placed on the build platform of a PolyJet^™^ printer, and the position of the anatomical variation influences the dimensional accuracy of the 3DPT. To accept the null hypothesis, no statistically significant differences were expected across the four analyzed variables—canal volume and centroids (X, Y, and Z). This condition was fulfilled only between certain groups (XA–YA and XB–YB), whereas significant differences were observed among the remaining groups. Therefore, the null hypothesis was only partially supported. These findings indicate that the printing orientation axis itself does not have a significant effect, whereas the position of the anatomical variation regarding the build platform does. This is evidenced by the absence of statistically significant differences between groups XA and YA, and likewise between groups XB and YB, although significant statistical differences were observed between positions A and B within the same axis. These findings are consistent with previous research showing that PolyJet™ printing exhibits dimensional variability influenced by the object’s orientation relative to the printhead [4,6,9,13,21], the printing material, and the geometry of the printed structure [22].

Nonetheless, an important question arises regarding the effectiveness of the support-material removal protocol described by Reis et al. 2024. Although the support material used (SUP706) is highly soluble in NaOH, allowing it to be removed easily and without mechanically stressing the printed parts and its dissolution does not compromise the dimensional accuracy of the samples [8], it is uncertain whether this protocol can totally remove the support material from regions as narrow as the anatomical variation analyzed (isthmus), or whether it may influence canal morphology, as previously discussed by those authors [5]. The protocol requires maintaining canal patency through passive insertion of a #15 K-file, which must be used five times in each canal. If patency was lost after these mandatory insertions, it was reestablished with the same file, a situation that occurred only in groups ZA and ZB. Although inserted passively, this resulted in an unequal number of #15 K-file insertions among canals, and its potential impact on root canal anatomy must be considered. Nonetheless, it is important to note that although these groups underwent a higher number of K-file insertions, they simultaneously exhibited the largest negative biases, demonstrating that their initial canal volumes were inherently smaller than those of the other groups. This indicates that the patency procedures have a neglectable effect on this variable. To eliminate this variability and isolate the effect of printing orientation and position, the canals were subsequently filled artificially, and analyses were performed on total tooth volume and area, which are unaffected by patency procedures.

In this study, the 3DPT showed significant differences compared with the NT and therefore could not be directly compared, in contrast to the results reported by Reis et al. 2024 who found no statistically significant differences between NT and 3DPT. However, as already mentioned, the present study used a tooth with anatomical variation, unlike the one used in their study [5]. Due to its smaller volume, this type of anatomical variation is more difficult to reproduce accurately. Previous studies have shown that models printed using the PolyJet™ technique when printed in high-quality mode may present dimensional expansion, as external features are generally produced slightly oversized, whereas internal features tend to be undersized [2,5,21,22,23,24]. Dimensional accuracy is strongly influenced by the polymerization rate and by the viscosity of the photocurable resin. The print head travels along the X-axis and applies material in a sequence of forward–backward movements, material is jetted only during the initial forward motion, while the subsequent movements serve exclusively to finalize the polymer curing. In this way, during printing, each newly deposited layer can flow slightly over the surface before full curing, explaining the dimensional expansion. In the present study, evaluation of the absolute and relative biases together with the corresponding LoA showed that all groups exhibited systematic deviations from the NT. Canal volume and centroid coordinates demonstrated negative biases, indicating consistent underestimation of internal features, whereas total tooth volume and area showed positive biases, reflecting systematic overestimation of external features. The 95% LoA widened progressively toward the Z-axis orientations and, importantly, were consistently broader in all B positions than in the corresponding A positions within the same axis. This pattern indicates that position on the build platform exerts a stronger influence than printing orientation axis on dimensional accuracy of 3DPT.

Among all groups, XA and YA consistently showed the smallest absolute and percentage biases and the narrowest 95% LoA across variables, indicating closer agreement with the NT and lower CV compared with the remaining orientations and positions. This can be attributed to the resolution of the PolyJet™ technique in the XY plane (42.3 µm) compared with the layer thickness along the Z-axis (16 µm). Positioning the major axis of the anatomical variation parallel to the build platform—so that it is printed within the resolution XY plane, resulted in superior print quality compared with the perpendicular orientation used in groups XB and YB. In the latter, the minor axis of the anatomical variation was printed within the resolution of XY plane, which may have contributed to reduced precision. Therefore, fine anatomical details should ideally be oriented facing upward to maximize accuracy [4,7]. Concerning printing along the Z-axis, the results of the present study align with previous reports indicating that vertically printed objects generally exhibit the lowest dimensional accuracy [22]. When the normal deviation values were visualized using a color deviation map in Figure 4, they generally fell within the manufacturer’s stated construction accuracy. According to the manufacturer, construction accuracy for Stratasys Objet30 Prime™ printer (Stratasys Ltd., Eden Prairie, MN, USA) is 0,1 mm, depending on the material used, model geometry and orientation, and printing parameters. This specification was mostly achieved except for groups ZA and ZB.

Horizontal printing tends to produce smoother surfaces, while vertical printing results in fine surface grooves which is reflected as a more pronounced staircase effect, as illustrated in Figure 4 and greater variability, resulting in higher CV values [2,4,8,9,10,12]. This behavior can be explained by the distinct mechanics of Z-axis and XY-axis fabrication. Along the Z-axis, layer positioning is controlled by a stepper-motor system, whereas in the XY plane the material is deposited directly by the printhead, which operates at approximately 600 dpi. With droplet sizes of about 30 µm, deviations of one to two droplets are possible. While such discrepancies become proportionally less relevant in larger printed objects, they appear comparatively more pronounced in small, detailed structures such as 3DPT [8].

Regarding the second aim of this study, comparing the final canal volume, alterations in the centroids X, Y, and Z, canal transportation, and percentage of untouched areas, it was found that there were no significant differences between the comparable groups (XA versus YA and XB versus YB). Therefore, the null hypothesis was accepted. The orientation in which the 3DPT is printed does not influence its behavior during endodontic preparation. In the present study, the percentage of canal volume increase ranged from 74.54% to 87.39%, while the literature reports values between 18.7% and 163.32% [5,20,25,26,27,28]. The percentage of unprepared area ranged from 34.28% to 38.53%, whereas in the literature it varies between 3.57% and 46.85% [5,20,25,26,27,28]. The differences between the results of this study and those reported in previous works can be attributed to the type of teeth or canals analyzed. Most studies, as in the present one, evaluated mandibular molars, which typically show higher percentages of canal volume increase and lower percentages of untouched area [20,25,27,28]. Variations in canal geometry directly affect the performance of preparation techniques [29]. In addition, in the present study, the 3DPT presented mesial canals that merged into a single canal in the apical third, resulting in this portion being prepared twice, which may have also influenced the obtained results.

This study has several limitations. First, only a single tooth with one specific anatomical variation was analyzed, and printing was performed using a single PolyJet™ printer. Multiple factors inherent to this technology can influence the final printed geometry. For example, nozzle blockage or printhead contamination may impair the deposition of photopolymer, reduce material flow and cause measurable decreases in printed parts dimensions. Furthermore, machine warm-up effects may introduce further variability: because the material is jetted at approximately 73 °C onto a build platform at ambient temperature, the resulting thermal gradient exceeding 50 °C can induce localized residual stresses within the deposited layers, limiting normal contraction as the material cools upon contact with the substrate [2,12]. In addition, only one printing resin and one type of support material were used, although it is well established that 3D printing outcomes are material dependent [8]. Despite these limitations, the findings indicate that the tooth should be positioned with its major root axis in cross-section as parallel as possible to the build platform. Similarly, when printing teeth with anatomical variations, the major axis of the variation should also be oriented parallel to the build platform to optimize accuracy. Future research should explore different anatomical configurations—such as lateral or recurrent canals—to further assess how these variations affect the printing behavior and dimensional accuracy of PolyJet™-produced 3DPT.

It is well established that improving the quality of printed models depends on understanding the interaction between material behavior, process parameters, and the resulting structural characteristics. This relationship is essential for taking full advantage of 3D-printing technologies [12]. Considering the findings of the present study, we recommend that future research using PolyJet™-printed 3DPT conduct preliminary evaluations—tailored to the anatomical features being studied—to identify the most suitable positioning of the tooth on the build platform. Such optimization will enable the fabrication of 3DPT that more accurately replicate NT morphology. The present findings also highlight that, by carefully defining the printing and methodological parameters, an efficient experimental design for 3DPT testing can be established, enhancing 3DPT for endodontic research applications.

## 5. Conclusions

Within the limitations of this study, printing orientation and position on the build platform both influenced the dimensional accuracy of 3DPT produced using PolyJet™ technology; however, tooth position (A/B) exerted a stronger effect than tooth orientation printing axes X/Y. Teeth printed along the X and Y axes within the same position, showed similar behavior during canal preparation with ProTaper Gold^®^, indicating that orientation did not substantially affect endodontic preparation behavior, as measured by volume change, transportation, and untouched areas. Accurate positioning of the tooth, particularly with respect to its internal anatomy, and avoiding printing along the Z-axis are therefore key to optimizing PolyJet™ 3DPT, offering a standardized and reproducible alternative to NT for endodontic research.

## Figures and Tables

**Table 1:** Microcomputed tomographic analysis before preparation of natural tooth and 3D-printed teeth.

	Natural Tooth		3D Printed Teeth					
			XA	XB	YA	YB	ZA	ZB
Canal Volume (mm^3^)	12.40	Mean ± SD	10.23 ± 0.31	9.02 ± 0.81	10.58 ± 0.31	9.22 ± 0.36	8.44 ± 0.44	7.59 ± 0.86
		Median	10.28	9.38	10.54	9.15	8.37	7.70
		Min–Max	9.72–10.67	7.96–10.04	10.19–11.19	8.82–10.06	7.85–9.40	6.00–8.54
Centroid X (mm)	13.28	Mean ± SD	13.20 ± 0.03	13.13 ± 0.08	13.16 ± 0.05	13.13 ± 0.04	13.12 ± 0.06	13.19 ± 0.04
		Median	13.20	13.12	13.17	13.12	13.13	13.19
		Min–Max	13.15–13.25	13.03–13.32	13.02–13.22	13.03–13.22	12.99–13.19	13.14–13.26
Centroid Y (mm)	12.64	Mean ± SD	12.52 ± 0.04	12.42 ± 0.06	12.54 ± 0.02	12.45 ± 0.03	12.51 ± 0.05	12.50 ± 0.05
		Median	12.53	12.43	12.54	12.45	12.50	12.51
		Min–Max	12.45–12.58	12.32–12.51	12.49–12.57	12.38–12.49	12.43–12.57	12.41–12.56
Centroid Z (mm)	11.20	Mean ± SD	11.13 ± 0.06	11.05 ± 0.08	11.10 ± 0.05	11.02 ± 0.07	11.05 ± 0.11	10.89 ± 0.08
		Median	11.12	11.07	11.10	11.03	11.05	10.93
		Min–Max	11.07–11.25	10.90–11.16	11.02–11.20	10.90–11.11	10.82–11.19	10.77–10.99
Total Tooth Volume (mm^3^)	381.45	Mean ± SD	387.56 ± 3.23	385.55 ± 4.31	389.27 ± 3.19	391.10 ± 3.96	391.10 ± 3.91	396.45 ± 4.77
		Median	387.40	386.27	389.87	390.31	390.38	396.19
		Min–Max	381.75–392.30	378.20–393.45	384–393.54	386.90–398.51	385.53–398.64	389.07–403.15
Total Tooth Area (mm^2^)	430.00	Mean ± SD	454.24 ± 2.85	465.30 ± 5.21	450.72 ± 2.65	463.01 ± 4.55	500.76 ± 8.15	512.41 ± 11.69
		Median	454.16	463.97	451.11	462.18	500.90	518.71
		Min–Max	450.60–460.84	459.86–475.78	445.82–454.30	457.65–469.99	488.93–513.79	492.84–523.89

**Table 2:** Absolute (mm, mm^2^, mm^3^) and relative (%) biases of 3D printed teeth groups compared with the natural tooth and corresponding 95% limits of agreement (95% LoA).

		3D Printed Teeth					
		XA	XB	YA	YB	ZA	ZB
Canal Volume (mm^3^)	Bias	−2.17	−3.38	−1.83	−3.18	−3.96	−4.81
	95% LoA	−2.78 to −1.56	−4.97 to −1.79	−2.44 to −1.22	−3.89 to −2.47	−4.82 to −3.10	−6.50 to −3.12
Centroid X (mm)	Bias	−0.08	−0.15	−0.12	−0.15	−0.16	−0.09
	95% LoA	−0.14 to −0.02	−0.31 to 0.01	−0.22 to −0.02	−0.23 to −0.07	−0.28 to −0.04	−0.17 to −0.01
Centroid Y (mm)	Bias	−0.12	−0.22	−0.10	−0.19	−0.14	−0.14
	95% LoA	−0.20 to −0.04	−0.34 to −0.10	−0.14 to −0.06	−0.25 to −0.13	−0.24 to −0.04	−0.24 to −0.04
Centroid Z (mm)	Bias	−0.07	−0.15	−0.10	−0.18	−0.15	−0.31
	95% LoA	−0.19 to 0.05	−0.31 to 0.01	−0.20 to 0.00	−0.32 to −0.04	−0.37 to 0.07	−0.47 to −0.15
Total Tooth Volume (mm^3^)	Bias	6.11	4.10	7.82	9.65	9.65	15.00
	95% LoA	−0.22 to 12.44	−4.35 to 12.55	1.57 to 14.07	1.89 to 17.41	1.99 to 17.31	5.65 to 24.35
Total Tooth Area (mm^2^)	Bias	24.24	35.30	20.72	33.01	70.76	82.41
	95% LoA	18.65 to 29.83	25.09 to 45.51	15.53 to 25.91	24.09 to 41.93	54.79 to 86.73	59.50 to 105.32
Canal Volume (%)	Bias	−17.52	−27.24	−14.78	−25.66	−31.94	−38.80
	95% LoA	−22.48 to −12.56	−40.06 to −14.42	−19.72 to −9.84	−31.42 to −19.90	−38.82 to −25.06	−52.38 to −25.22
Centroid X (%)	Bias	−0.59	−1.11	−0.90	−1.11	−1.22	−0.66
	95% LoA	−1.10 to −0.08	−2.31 to 0.09	−1.70 to −0.10	−1.76 to −0.46	−2.08 to −0.36	−1.31 to −0.01
Centroid Y (%)	Bias	−0.07	−1.72	−0.81	−1.53	−1.07	−1.12
	95% LoA	−0.19 to 0.05	−2.66 to −0.78	−1.16 to −0.46	−2.04 to −1.02	−1.81 to −0.33	−1.96 to −0.28
Centroid Z (%)	Bias	−0.60	−1.31	−0.88	−1.64	−1.35	−2.74
	95% LoA	−1.58 to 0.38	−2.64 to 0.02	−1.74 to −0.02	−2.82 to −0.46	−3.19 to −0.49	−4.15 to −1.33
Total Tooth Volume (%)	Bias	1.60	1.08	2.05	2.53	2.53	3.93
	95% LoA	−0.07 to −3.27	−1.13 to 3.29	0.40 to 3.70	0.51 to 4.55	0.53 to 4.53	1.48 to 6.38
Total Tooth Area (%)	Bias	5.64	8.21	4.82	7.68	16.46	19.16
	95% LoA	4.35 to 6.93	5.84 to 10.58	3.60 to 6.04	5.60 to 9.76	12.74 to 20.18	13.83 to 24.49

**Table 3:** Coefficient of variation in Total tooth Volume and Total Tooth Area for each 3D-printed teeth group.

		3D Printed Teeth					
		XA	XB	YA	YB	ZA	ZB
Total Tooth Volume (mm^3^)	Mean ± SD	387.56 ± 3.23	385.55 ± 4.31	389.27 ± 3.19	391.10 ± 3.96	391.10 ± 3.91	396.45 ± 4.77
	Coefficient of variation	0.83	1.12	0.82	1.01	1.00	1.20
Total Tooth Area (mm^2^)	Mean ± SD	454.24 ± 2.85	465.30 ± 5.21	450.72 ± 2.65	463.01 ± 4.55	500.76 ± 8.15	512.41 ± 11.69
	Coefficient of variation	0.63	1.12	0.59	0.98	1.63	2.28

**Table 4:** Microcomputed tomographic analysis of groups XA versus YA, and XB versus YB before and after preparation with ProTaper Gold^®^.

			XA	YA	XB	YB
Canal Volume (mm^3^)	Initial	Mean ± SD	10.23 ± 0.31	10.58 ± 0.31	9.02 ± 0.81	9.22 ± 0.36
		Min–Max	9.72–10.67	10.19–11.19	7.96–10.04	8.82–10.06
	After	Mean ± SD	17.97 ± 0.52	18.25 ± 0.50	17.05 ± 0.58	16.63 ± 0.36
		Min–Max	17.20–18.73	17.21–18.83	16.32–18.22	16.19–17.49
	% Volume Increase	Mean ± SD	75.87 ± 7.20	72.87 ± 7.72	90.14 ± 16.12	80.18 ± 8.35
		Min–Max	63.54–86.34	53.80–81.94	71.79–112.27	63.12–90.82
Centroid X (mm)	Initial	Mean ± SD	13.20 ± 0.03	13.16 ± 0.05	13.13 ± 0.08	13.13 ± 0.04
		Min–Max	13.15–13.25	13.02–13.22	13.03–13.32	13.03–13.22
	After	Mean ± SD	13.66 ± 0.04	13.64 ± 0.03	13.60 ± 0.05	13.61 ± 0.03
		Min–Max	13.58–13.72	13.59–13.70	13.52–13.70	13.56–13.68
	Deviation	Mean ± SD	0.46 ± 0.06	0.48 ± 0.06	0.46 ± 0.12	0.48 ± 0.04
		Min–Max	0.39–0.54	0.41–0.63	0.20–0.58	0.41–0.54
Centroid Y (mm)	Initial	Mean ± SD	12.52 ± 0.04	12.54 ± 0.02	12.42 ± 0.06	12.45 ± 0.03
		Min–Max	12.45–12.58	12.49–12.57	12.32–12.51	12.38–12.49
	After	Mean ± SD	12.99 ± 0.03	13.02 ± 0.02	12.93 ± 0.06	12.99 ± 0.05
		Min–Max	12.92–13.02	12.98–13.07	12.86–13.03	12.95–13.11
	Deviation	Mean ± SD	0.46 ± 0.06	0.49 ± 0.03	0.51 ± 0.08	0.53 ± 0.04
		Min–Max	0.34–0.55	0.42–0.54	0.42–0.62	0.48–0.61
Centroid Z (mm)	Initial	Mean ± SD	11.13 ± 0.06	11.10 ± 0.05	11.05 ± 0.08	11.02 ± 0.07
		Min–Max	11.07–11.25	11.02–11.20	10.90–11.16	10.90–11.11
	After	Mean ± SD	11.44 ± 0.04	11.45 ± 0.04	11.45 ± 0.05	11.40 ± 0.04
		Min–Max	11.35–11.48	11.38–11.53	11.34–11.50	11.35–11.48
	Deviation	Mean ± SD	0.30 ± 0.08	0.35 ± 0.08	0.39 ± 0.08	0.37 ± 0.06
		Min–Max	0.20–0.41	0.23–0.45	0.26–0.57	0.28–0.47
Canal Transportation (mm)	Mean ± SD		0.72 ± 0.08	0.77 ± 0.06	0.80 ± 0.10	0.82 ± 0.08
	Min–Max		0.57–0.84	0.66–0.86	0.64–0.94	0.73–0.97
Unprepared area (%)	Mean ± SD		36.93 ± 2.99	38.18 ± 3.82	35.37 ± 5.48	38.86 ± 5.01
	Min–Max		32.89–42.24	31.96–44.58	28.59–49.29	31.86–49.98

## Data Availability

The data that supports the findings of this study are available from the corresponding author upon reasonable request. The data is not publicly available due to privacy and ethical restrictions (undergoing PhD thesis).

## Abbreviations

The following abbreviations are used in this manuscript:

3D	Three-dimensional
NT	Natural teeth
Micro-CT	Micro-computed tomography
PTG	ProTaper Gold®
STL	Stereolithography
SD	Standard deviation
LoA	Limit of agreement
CV	Coefficient of variation
